# Interventions for the prevention of risk factors and incidence of type 2 diabetes in the work environment: a systematic review

**DOI:** 10.11606/s1518-8787.2019053001084

**Published:** 2019-11-18

**Authors:** Jorge Inolopú, Nélida Hilario-Huapaya, Martín Alonso Tantaleán-Del-Águila, Yamilee Hurtado-Roca, Cesar Ugarte-Gil

**Affiliations:** I Universidad Peruana Cayetano Heredia. Instituto de Medicina Tropical Alexander von Humboldt. Lima, Perú; II Universidad Peruana Cayetano Heredia. Facultad de Salud Pública. Lima, Perú; III EsSalud. Subgerencia de Atención Preventivo Promocional y Complejidad Creciente. Lima, Perú; IV EsSalud. Instituto de Evaluación de Tecnologías en Salud e Investigación. Lima, Perú; V Universidad Peruana Cayetano Heredia. Facultad de Medicina. Lima, Perú

**Keywords:** Type 2 Diabetes Mellitus, prevention & control, Assessment, Outcome (Health Care), Working Environment, Review

## Abstract

**OBJECTIVE:**

To evaluate the effectiveness of interventions aimed at the prevention of risk factors and incidence of type 2 diabetes in the workers population.

**METHODS:**

Systematic review of interventions aimed at adult workers at risk of type 2 diabetes published in Medline, Embase, Web of Science, Central Cochrane Registry of Controlled Trials, and Lilacs. Randomized trials, quasi-experimental research and cohort studies were selected; in English, Spanish and Portuguese; published from 2000 to 2017. Intervention effectiveness was evaluated concerning the incidence of type 2 diabetes and a significant reduction in body weight, or another anthropometric or metabolic parameter.

**RESULTS:**

3,024 articles were generated, of which 2,825 that did not answer the research question were eliminated, as well as 130 that did not evaluate original interventions, 57 carried out outside the workplace and two reviews; so that 10 selected items remained. Interventions based on structured programs previously evaluated and integrated into the workplace had a favorable impact on the reduction of body weight and other risk factors.

**CONCLUSIONS:**

The effectiveness of lifestyle interventions for the prevention of type 2 diabetes should be based on structured programs with proven effectiveness and adapted to the workplace, with employer participation in the provision of schedules and work environments.

## INTRODUCTION

Type 2 diabetes (DT2) is estimated to affect 425 million people, which represent 8.8% of the world’s adult population^1^ . The numbers are expected to increase to 693 million by 2045^2^ . The prevention of DM2 through structured programs to promote physical activity and healthy diet, also known as lifestyle interventions (LSI), have proved to be effective in reducing the risk of DM2^3 , 4^ . The Finnish Diabetes Prevention Study (FDPS)^5^ and the Diabetes Prevention Program (DPP)^6^ propose as a success indicator the reduction of 5.0 and 7.0% of the initial body weight, respectively. However, the success and sustainability of these interventions also depends on the context in which they are developed^7^ . Work environment represents a challenge for LSIs, considering its known risk factors, such as inadequate diet^8 , 9^ and sedentary lifestyle^10 , 11^ , added to other yet uunevaluated factors, such as work stress^12^ . The prevention of DM2 and its complications in labor contexts aims to maintain workers’ health and well-being, in addition to avoiding absenteeism^13^ , low productivity^14^ and significant expenses that affect the health system^15 , 16^ . This study aimed to evaluate the effectiveness of interventions for the prevention of DM2 risk factors in the worker population.

## METHODS

A systematic review of interventions aimed at an adult worker population at risk of DM2 was performed. Original articles in English, Spanish and Portuguese, designed as randomized trial, quasi-experimental or cohort studies, and published between 2000 and 2017 were considered eligible. Such studies included interventions for the prevention of DM2 risk factors, based or not on a previously evaluated program. The study population included adult workers with risk factors for DM2, such as overweight, obesity, pre-diabetes and/or metabolic syndrome. Those with some type of diabetes at the beginning of intervention were excluded.

A highly sensitive search algorithm was used for the PubMed, Embase, Web of Science, and Lilacs databases and in the Central Cochrane Register of Controlled Trials (CENTRAL) ( Chart ).

**Chart:** Search terms.

•Pubmed: ((“Cohort Studies”[Mesh] OR “Clinical Trial” [Publication Type]) AND ((“2000/01/01”[Date - Publication] : “2017/30/12”[Date - Publication]))) AND (“Adult”[Mesh] OR “Occupational Groups”[Mesh] OR “Occupational Health Nursing”[Mesh] OR “Workplace”[Mesh] OR (“Occupational Groups”[Mesh] AND “Population”[Mesh]) OR Work-related[All Fields] OR Worksite[All Fields] OR Working population[All Fields] OR Economically active population[All Fields]) AND (“Occupational Health”[Mesh] OR “Primary Prevention”[Mesh] OR “Occupational Health Services”[Mesh] OR “Preventive Health Services”[Mesh] OR “Complementary Therapies”[Mesh] OR “Integrative Medicine”[Mesh] OR “Models, Organizational”[Mesh] OR “Behavior Therapy”[Mesh] OR “Tai Ji”[Mesh] OR “Yoga”[Mesh] OR “Qigong”[Mesh]) AND (“Diabetes Mellitus, Type 2” [Mesh]) AND (Diabetes Mellitus, Type 2/prevention and control[MAJR] OR “Prediabetic State”[Mesh] OR “Hyperglycemia”[Mesh] OR “Obesity, Abdominal”[Mesh] OR “Obesity”[Mesh] OR “Hypertension”[Mesh] OR “Metabolic Syndrome X”[Mesh] OR “Overweight”[Mesh] OR “Body Mass Index”[Mesh] OR “Body Weight”[Mesh] OR “Sagittal Abdominal Diameter”[Mesh] OR “Self Care”[Mesh] OR “Health Knowledge, Attitudes, Practice”[Mesh] OR “Quality of Life”[Mesh]) NOT “Diabetes, Gestational”[Mesh] NOT “Animal Experimentation”[Mesh] NOT “Diabetes Mellitus, Type 1”[Mesh]
•Embase: #1: (‘prevention’/exp OR ‘yoga’/exp OR ‘tai chi’/exp OR ‘alternative medicine’/exp OR ‘primary prevention’/exp OR ‘occupational health’/exp); #2: (‘diabetes mellitus’/exp OR ‘impaired glucose tolerance’/exp OR ‘obesity’/exp OR ‘metabolic syndrome x’/exp) AND (‘prevention’/exp OR ‘yoga’/exp OR ‘tai chi’/exp OR ‘alternative medicine’/exp OR ‘primary prevention’/exp OR ‘occupational health’/exp); #3: (‘cohort analysis’/exp OR ‘cohort analysis’ OR [cochrane review]/lim OR [systematic review]/lim OR [meta analysis]/lim OR [controlled clinical trial]/lim OR [randomized controlled trial]/lim) AND (‘diabetes mellitus’/exp OR ‘impaired glucose tolerance’/exp OR ‘obesity’/exp OR ‘metabolic syndrome x’/exp); #4: ([english]/lim OR [portuguese]/lim OR [spanish]/lim) AND [adult]/lim AND [humans]/lim AND [2000-2017]/py AND [article]/lim AND ([embase]/lim OR [embase classic]/lim); #5: #1 AND #2 AND #3 AND #4
•Web of Science: #1: (TS=(“Occupational Health” OR “Primary Prevention” OR “Occupational Health Services” OR “Preventive Health Services” OR “Complementary Therapies” OR “Integrative Medicine” OR “Models, Organizational” OR “Behavior Therapy” OR “Tai Ji” OR “Yoga” OR “Qigong”)); #2: (TS=(“diabetes” OR “Metabolic Syndrome” OR “Prediabetic State”)); #3: (TS=((“adult population” OR “Adult” OR “Occupational Groups” OR “Occupational Health Nursing” OR “Workplace” OR (“Occupational Groups” AND “Population”) OR “Work-related” OR “Worksite” OR “Working population” OR “Economically active population”))) AND LANGUAGE: (English OR Portuguese OR Spanish) AND DOCUMENT TYPES: (Article); #4: #1 AND #2 AND #3, Indexes=SCI-EXPANDED, SSCI, A&HCI, CPCI-S, CPCI-SSH, BKCI-S, BKCI-SSH, ESCI, CCR-EXPANDED, IC Timespan=2000-2017
•CENTRAL: #1: MeSH descriptor: [Diabetes Mellitus, Type 2] explode all trees and with qualifier(s): [Prevention & control - PC]; #2: MeSH descriptor: [Workplace] explode all tres; #3: #1 AND #2; Publication Year from 2000 to 2017
•Lilacs: (tw:(DIABETES)) AND (tw:(PREVENTION)) AND (tw:(WORKPLACE))

A list of titles and abstracts was created, based on results of the database search with a bibliographic reference manager (EndNote ^TM^ x8, USA). The selection strategy included a first screening of titles and/or abstracts and a second screening based on full-text reading by two of the authors (JI and NH), resolving the discrepancies of both steps with a third reviewer (CU-G).

**Figure 1:**
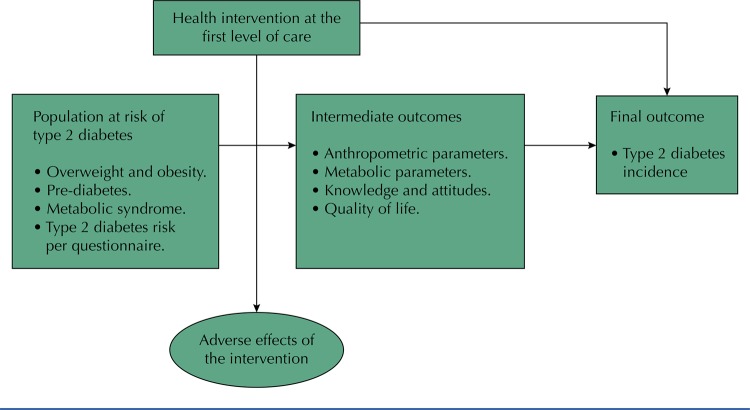
Framework of the systematic review.

Intervention effectiveness considered the incidence of DM2 or a significant reduction of initial body weight, as this parameter is recognized as the main DM2 risk predictor^17 , 18^ , as well as of any other anthropometric or metabolic parameter recognized as a DM2 risk predictor ( Figure 1 ).

## RESULTS

**Figure 2:**
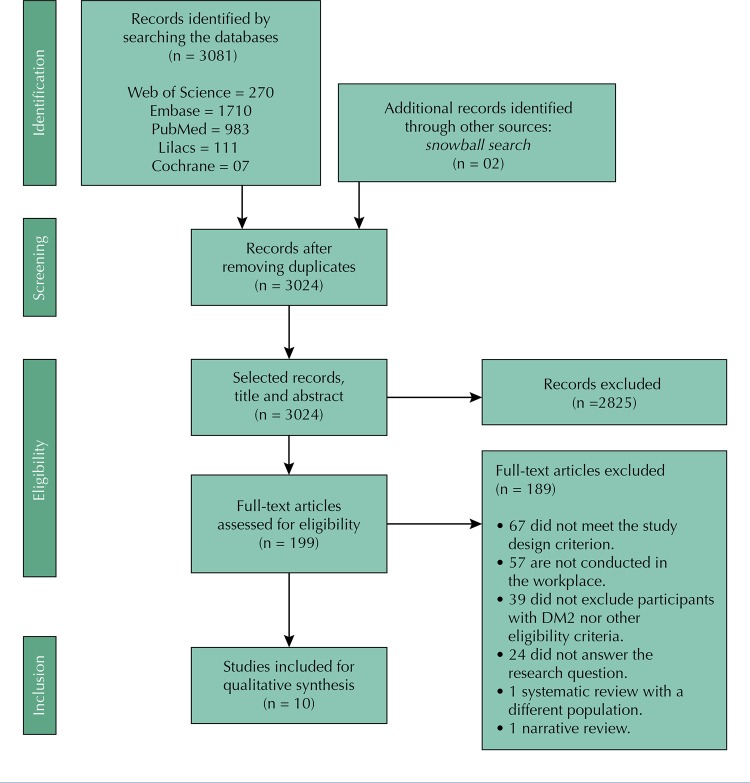
Selection process of studies included in the analysis.

The search generated 3,083 articles, of which 59 duplicate articles were eliminated, leaving 3,024. In the first screening, 2,825 articles that did not answer the research question were excluded. In the second screening, 189 articles were excluded because they had another study design (n = 67), did not consider the work environment (n = 57) and did not exclude participants with DM2 (n = 39). Therefore, 10 articles were selected, of which six were randomized trials and four were quasi-experimental studies testing the before and after of a single group ( Figure 2 ). Results show heterogeneity in the number, duration, and content of intervention sessions evaluated, as well as diverse age groups, educational levels and work environments of the participants ( Table 1 ).

**Table 1:** Characteristics of studies on the prevention of DM2 risk factors in the work environment.

Reference	Country	Intervention - - Program	Study design	Follow-up (months)	Risk factor	Population	Field of work
Limaye et al.^25^ (2016)	India	LSI 2 - NICE	Randomized Trial	12	≥ 3 cardiometabolic risk factors	•265 participants: 132 in the control and 133 in the intervention group, with a mean of 36.2 years of age (SD = 8.0), being 75% male.	Computer science
Kramer et al.^19^ (2015)	USA	LSI 1 - DPP	Randomized Trial	12	Pre-diabetes, BMI ≥ 24, MS or hyperlipidemia	•89 participants: 60 individuals in the first group (advanced) and 29 in the second (late), with an average age of 52.3 years old (range: 34–70), being 45% male.	Pharmaceutical company
Weinhold et al.^20^ (2015)	USA	LSI 1 - DPP	Randomized Trial	07	Pre-diabetes + BMI ≥ 25	•69 participants (34 in control and 35 in intervention) from 18 and 65 years old, being 20% male.	University employees
Faghri et al.^21^ (2014)	USA	LSI 1 - DPP	Randomized Trial	07	Risk Score (DRS) + BMI ≥ 25	•99 participants: 51 in the group with incentive and 48 in the group without incentive. Average age of the group without and with incentive: 48.98 (SD = 11.23) and 45.14 (SD = 11.27) years old, respectively.	Nursing Technicians
Maruyama et al.^24^ (2010)	Japan	LSI 1 - LiSM10!	Randomized Trial	04	MS	•87 male participants: 49 in the control group and 52 in the intervention group, with an average of 52.0 (SD = 7.9) years old.	Clerks
Watanabe et al.^26^ (2003)	Japan	Nutritional - JDS/ADA	Randomized Trial	12	Pre-diabetes	•173 male participants: 77 in the control group and 79 in the intervention group, with an average of 55 years old.	Not specified
Zyriax et al.^22^ (2014)	Germany	LSI 1 - FDPS	Pre- and post-test study	36	Large abdominal circumference + pre-diabetes	•300 participants with an average age of 43.6 years old. Participants at DM2 risk and with DM2 diagnosis are evaluated separately.	Not specified
Viitasalo et al.^23^ (2015)	Finland	LSI 1 - FDPS	Pre- and post-test study	30	Risk score	•1347 participants: 945 at low risk of DM2 and 402 at a high risk of DM2, with an average age in men and women of 50 (SD = 7.3) and 47 (SD = 8.0) years old, respectively.	Airline employees
Burton et al.^2^ (2015)	USA	LSI 1 - HLDP	Pre- and post-test study	12	Pre-diabetes	•34 participants with pre-diabetes, 65 with diabetes and 2 with other risk factors. Participants at DM2 risk and with DM2 diagnosis are evaluated separately.	Financial services
Bevis et al.^28^ (2014)*	USA	LSI 2 - NR	Pre- and post-test study	12	Pre-diabetes	•73 participants with pre-diabetes, with an average age of 50.0 (SD = 10.0) years old, being 74% male. Participants at DM2 risk and with DM2 diagnosis are evaluated separately.	Not specified

LSI 1: conventional intervention (with a nutritional and physical activity approach); LSI 2: conventional intervention, with virtual monitoring and counseling; Nutritional: exclusively nutritional intervention; MS: metabolic syndrome; BMI: body mass index; DPP: Diabetes Prevention Program; FDPS: Finnish Diabetes Prevention Study; LiSM10!: Life Style Modification Program for Physical Activity and Nutrition Program; JDS/ADA: Japan Diabetes Society and American Diabetes Association; HLDP: Healthy Living with Diabetes Program; NR: Not reported. SD: Standard Deviation. DRS: Diabetic Retinopathy Study.

**Figure 3:**
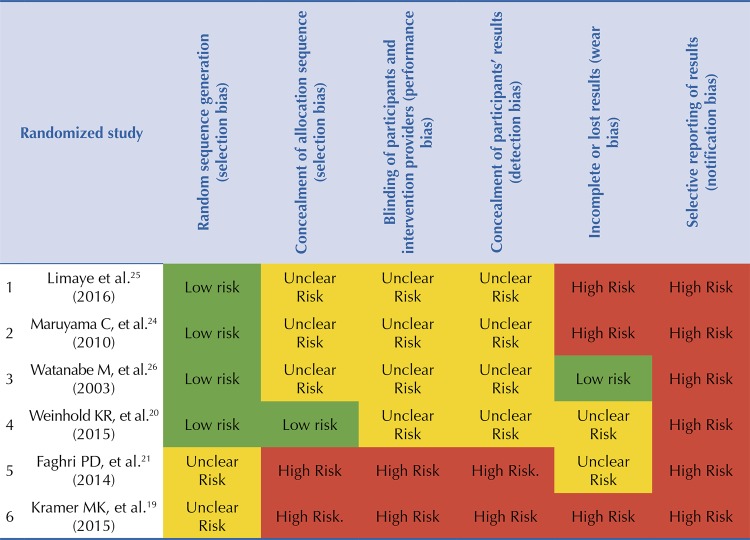
Summary of the bias risk assessment of randomized studies according to the Cochrane Collaboration.

Interventions were classified as: conventional LSI (seven), virtual counseling LSI (two), and exclusively nutritional (one). Of these, six were based on structured programs for the prevention of DM2, such as the DPP^19^ , FDPS^22 , 23^ and the Life Style Modification Program for Physical Activity and Nutrition program (LiSM10!)^24^ . Two interventions were ground on DM2 prevention recommendations based on guidelines from The National Institute for Health and Care Excellence (NICE)^25^ and the Japan Diabetes Society and the American Diabetes Association (JDS/ADA)^26^ . In addition, one intervention was based on a DM2 management program called Healthy Living with Diabetes Program^27^ and one did not report any background^28^ ( Table 2 ). Bias risk assessment was described and assessed as recommended by the Cochrane Collaboration for randomized studies^29^ ( Figure 3 ).

**Table 2:** Results of studies on the prevention of DM2 risk factors in the work environment.

Reference	Country	Intervention - structure	Incentive	Employer’s role	Outcome	Rating
Limaye et al.^25^ (2016)	India	1 group session at the beginning and every 3 months.	NR	Enrollment	•Significant reduction of body weight, BMI and abdominal circumference concerning the control group. Reduction of 6.0% in the prevalence of BMI ≥ 25 kg/m^2^ is reported in the intervention group, as well as a 6.8% increase in the control group. •No reduction in the percentage of weight lost is reported.	Effective
Kramer et al.^19^ (2015)	USA	12 weekly sessions for 3 months, 4 biweekly sessions for 3 months, and 6 monthly meetings of maintenance for 6 months.	Discount cards.	Work environment and schedules	•Significant reduction in body weight, HbA1C, abdominal perimeter and BMI between the study groups at 6 months of intervention. •Reduction of 5% of initial body weight by 45% in the first group and 7% in the second group at 6 months of intervention.	Effective
Weinhold et al.^20^ (2015)	USA	16 weekly sessions for 4 months.	NR	Work environment and schedules	•Significant reduction in weight, BMI, abdominal circumference, and fasting glucose levels in the intervention group and concerning the control group. •In the intervention and control group, 52.9% and 2.9% of the participants managed to lose at least 5% of body weight, respectively, in the post-intervention period.	Effective
Faghri et al.^21^ (2014)	USA	16 60-minute sessions per week, for 4 months.	Cumulative economic incentive.	Enrollment	•Significant reduction in weight and BMI in the group with economic incentive when compared to the group without economic incentive. •No reduction in the percentage of weight lost is reported.	Effective
Maruyama et al.^24^ (2010)	Japan	1 session at the beginning of intervention, individual counseling for 2 months, and online counseling for the following 2 months.	NR	Enrollment	•Significant reduction of 14 out of 17 parameters that define the risk of developing DM2, among which are weight and body mass index. •No reduction in the percentage of weight lost is reported.	Effective
Watanabe et al.^26^ (2003)	Japan	1 session per month of study and 1 session at the sixth month of study, via e-mail.	NR	Not specified	•Significant reduction in daily caloric intake, especially at night, with subsequent decrease in the 2-hour postprandial glucose levels when compared to the control group. •No reduction in the percentage of weight lost is reported.	Effective
Zyriax et al.^22^ (2014)	Germany	6 monthly sessions for 6 months, 6 biweekly sessions for 6 months, and 1 session every four months for 2 years.	NR	Enrollment	•Significant reduction in weight, body mass index, and abdominal circumference. In men and women there was a reduction of 4.3 ± 3.6 and 3.6 ± 2.4 kg, respectively, in the post-intervention period. •No reduction in the percentage of weight lost is reported.	Effective
Viitasalo et al.^23^ (2015)	Finland	Not specified.	NR	Enrollment	•There was an increase in the risk of DM2, while in the high-risk group the reduction in DM2 risk was not significant. •Reduction of ≥ 5% was reported in 20.8% of men and 7.0% of women who attended more than one session.	Uneffective
Burton et al.^27^ (2015).	USA	Not specified.	Glucometers and economic incentive when completing the study.	Wellness program	•There was no significant reduction in Body Mass Index (BMI) during the study, which was shown at levels greater than 30 kg/m^2^ during the study. •No reduction in the percentage of weight lost is reported.	Uneffective
Bevis et al.^28*^ (2014)	USA	4 2-hour educational sessions in the first semester and 8 subsequent telephone sessions.	Glucometers and affiliation in wellness program.	Wellness program	•There was no significant reduction in BMI, which was greater than 35 kg/m^2^ during the study, nor was there a significant reduction in glycosylated hemoglobin levels (HbA1c). •No reduction in the percentage of weight lost is reported.	Uneffective

NR: not reported; BMI: body mass index; DM2: type 2 diabetes; HbA1c: glycosylated hemoglobi

According to the effectiveness assessment, six studies showed statistically significant reduction in bodyweight^20^ , all of which were based on structured programs for the prevention of DM2, such as DPP^19 - 21^ , FDPS^22^ and LiSM10!^24^ , as well as on the recommendations given by NICE^25^ . On the other hand, a nutritional intervention showed a reduction in caloric intake with subsequent reduction of two-hour postprandial glucose levels, based on the JDS/ADA recommendations^26^ . In contrast, two non-effective interventions focused on the management of DM2^27 , 28^ , and one had limited sessions and prolonged follow-up^23^ . No studies showed adverse effects in the study population.

None of the seven effective studies showed results in terms of reducing the risk of DM2 incidence, since their design did not allow it. However, two of them showed results regarding the percentage of initial body weight reduction^19 , 20^ . Kramer et al.^19^ determined that 5.0% reduction of average body weight was achieved by 45.0% and 7.0% of the participants involved in the intervention and the control group, respectively^19^ . In contrast, Weinhold et al.^20^ determined that 32.4% of the intervention group managed to lose at least 7.0% of the initial body weight, a percentage which was significantly greater than for the control group (2.9%; p <0.01).

On the other hand, they also evaluated economic and non-economic incentives, as well as the role played by the employer’s participation in the intervention results. Faghri et al.^21^ was the only study to offer a cumulative economic incentive for lost weight. Despite being a low intensity intervention, it was successful in reducing weight, decreasing the risk of DM2 and leading to a healthy diet. Employer participation generally did not go further than supporting the enrollment of participants. The facilities in enrollment, arrangement of environments, and work schedules in the studies of Kramer et al.^19^ and Weinhold et al.^20^ were linked to the success of the intervention.

## DISCUSSION

Interventions based on structured programs for the prevention of DM2 showed wide effectiveness, which was linked to the degree of similarity with the base program. On the other hand, interventions based on recommendations from the NICE guidelines were statistically effective, but present aspects to be considered. The study by Limaye et al.^25^ was based on the NICE guideline and shows significant weight reduction, but this is achieved due to an increase in weight in the control group. In this study, the average bodyweight reduction in the intervention group does not reach less than 5.0% post-intervention.

Uneffective interventions^23 , 27 , 28^ did not have a structured session plan^23^ , and two of them were focused on the management of DM2^27 , 28^ . Viitasalo et al.^23^ , in a pre- and post-test study carried out on employees of an airline company, showed a slight increase in anthropometric parameters after the intervention. Such intervention had an average of 1.6 sessions, which is less than in any other existing program; although based on the FDPS, the program differed from the original. Regarding the two interventions aimed at DM2 management, they included a pre-diabetes population in a context of workers with DM2. These types of interventions were based on work welfare programs and, despite having resources, the intervention approach is important.

Overall, the selected articles make a brief description of the labor factors that would favor the development of DM2. Limaye et al.^25^ evaluated an intervention in IT employees in India who reported a sedentary lifestyle, inadequate eating habits, and work stress. Maruyama et al.^24^ evaluated Japanese office employees with prolonged periods of work, which would favor a high caloric intake. In contrast, Watanabe et al.^26^ , evaluated employees with working conditions related to high caloric intake at night and short periods of sleep.

Employees who carried out these activities are within the framework of “white-collar workers”. This population has a higher risk of being overweight and obese due to the sedentary lifestyle they experience at work, favoring the increase in body mass index^10^ . On the other hand, work with night, rotating shifts, or non-regular, called “shift work,” are associated with the development of DM2 due to insufficient hours of sleep and poor eating habits^31^ . Nonetheless, these factors were not evaluated in the intervention, but they had to be considered in the evaluation of its impact.

The employer’s participation in the enrollment was given by telephone calls and emails^21 , 23 - 25^ . Other studies also developed communication plans to encourage changes in lifestyle^19 , 20 , 22 , 27 , 28^ and even provided environments and schedules for the development of work sessions^19 , 20 , 27^ . Some interventions provide basic incentives such as glucometers^27 , 28^ , discount cards when initiating and completing the intervention ^19 , 27^ , and economic incentives for lost weight^21^ . However, the long-term results regarding the maintenance of the weight lost by this type of interventions seem to be inconsistent^32^ .

Those employers who offered greater support in the intervention, such as facilities in enrollment, provision of environments (topical, rest environments, coffee shops, etc.) and schedules at work favored the implementation and success of the intervention^19 , 20^ . Such benefits can be integrated to the intervention, to achieve and maintain the proposed objectives. Companies with food service can implement healthy menu options for people at risk of DM2, and even for diabetics^33^ . In our review, interventions that do not report the presence of a program with such benefits are those aimed primarily at “white-collar workers”^24 - 26^ .

The limitations of our review are linked to the heterogeneity of the interventions, which makes meta-analysis impossible. On the other hand, there are no results in terms of DM2 incidence risk due to the short intervention periods of most studies evaluated. Thus, the evaluation of intervention effectiveness is flexible, as they are based on the statistically significant reduction of body weight (p <0.05), but not on the reduction of body weight percentage or on clinically-significant weight reduction (reduction of at least 5.0% post-intervention). In addition, the risk of bias in randomized trials is high. The studies of Weinhold et al.^20^ and Watanabe et al.^26^ show a higher proportion of unclear risk, but with a better rating, while the article by Kramer et al.^19^ has the lowest rating.

Interventions with a favorable impact on the reduction of body weight and other risk factors of DM2 in the workplace are mainly those based on a program previously evaluated and carried out in the workplace with employer participation. Longitudinal studies are required to evaluate these interventions as potential programs to reduce the incidence of DM2 in the workplace.

## References

[1] 1. International Diabetes Federation. IDF Diabetes Atlas. 8. ed. Brussels (BEL): IDF; 2017 [citado 17 ene 2019]. Disponible en: http://www.diabetesatlas.org/

[2] 2. Cho NH, Shaw JE, Karuranga S, Huang Y, Fernandes JDR, Ohlrogge AW, et al. IDF Diabetes Atlas: global estimates of diabetes prevalence for 2017 and projections for 2045. Diabetes Res Clin Pract. 2018;138:271-81. 10.1016/j.diabres.2018.02.023 29496507

[3] 3. Tuomilehto J, Lindström J, Eriksson JG, Valle TT, Hämäläinen H, Ilanne-Parikka P, et al. Prevention of type 2 diabetes mellitus by changes in lifestyle among subjects with impaired glucose tolerance. N Engl J Med. 2001;344(18):1343-50. 10.1056/NEJM200105033441801 11333990

[4] 4. Diabetes Prevention Research Group. Reduction in the incidence of type 2 diabetes with lifestyle intervention or metformin. N Engl J Med. 2002;346(6):393-403. 10.1056/NEJMoa012512 PMC1370926 11832527

[5] 5. Eriksson J, Lindström J, Valle T, Aunola S, Hämäläinen H, Ilanne-Parikka P, et al. Prevention of Type II diabetes in subjects with impaired glucose tolerance: the Diabetes Prevention Study (DPS) in Finland. Study design and 1-year interim report on the feasibility of the lifestyle intervention programme. Diabetologia. 1999;42(7):793-801. 10.1007/s001250051229 10440120

[6] 6. Diabetes Prevention Program (DPP) Research Group. The Diabetes Prevention Program (DPP): description of lifestyle intervention. Diabetes Care. 2002;25(12):2165-71. 10.2337/diacare.25.12.2165 PMC1282458 12453955

[7] 7. Dunkley AJ, Bodicoat DH, Greaves CJ, Russell C, Yates T, Davies MJ, et al. Diabetes prevention in the real world: effectiveness of pragmatic lifestyle interventions for the prevention of type 2 diabetes and of the impact of adherence to guideline recommendations: a systematic review and meta-analysis. Diabetes Care. 2014;37(4):922-33. 10.2337/dc13-2195 24652723

[8] 8. Bonnell EK, Huggins CE, Huggins CT, McCaffrey TA, Palermo C, Bonham MP. Influences on dietary choices during day versus night shift in shift workers: a mixed methods study. Nutrients. 2017;9(3):193. 10.3390/nu9030193 PMC5372856 28245625

[9] 9. Nobrega S, Champagne N, Abreu M, Goldstein-Gelb M, Montano M, Lopez I, et al. Obesity/overweight and the role of working conditions: a qualitative, participatory investigation. Health Promot Pract. 2016;17(1):127-36. 10.1177/1524839915602439 PMC5860907 26333770

[10] 10. Lin TC, Courtney TK, Lombardi DA, Verma SK. Association between sedentary work and BMI in a U.S. National Longitudinal Survey. Am J Prev Med. 2015;49(6):e117-23. 10.1016/j.amepre.2015.07.024 26437869

[11] 11. Choi BK, Schnall PL, Yang H, Dobson M, Landsbergis P, Israel L, et al. Sedentary work, low physical job demand, and obesity in US workers. Am J Ind Med. 2010;53(11):1088-101. 10.1002/ajim.20886 20737422

[12] 12. Lian Y, Sun Q, Guan S, Ge H, Tao N, Jiang Y, et al. Effect of changing work stressors and coping resources on the risk of type 2 diabetes: The OHSPIW Cohort Sudy. Diabetes Care. 2018;41(3):453-60. 10.2337/dc17-0749 29255061

[13] 13. Ramsey S, Summers KH, Leong SA, Birnbaum HG, Kemner JE, Greenberg P. Productivity and medical costs of diabetes in a large employer population. Diabetes Care. 2002;25(1):23-9. 10.2337/diacare.25.1.23 11772896

[14] 14. Von Korff M, Katon W, Lin EHB, Simon G, Ciechanowski P, Ludman E, et al. Work disability among individuals with diabetes. Diabetes Care. 2005;28(6):1326-32. 10.2337/diacare.28.6.1326 15920047

[15] 15. Seuring T, Archangelidi O, Suhrcke M. The economic costs of Type 2 Diabetes: a global systematic review. Pharmacoeconomics. 2015;33(8):811-31. 10.1007/s40273-015-0268-9 PMC4519633 25787932

[16] 16. Arredondo A. Type 2 diabetes and health care costs in Latin America: exploring the need for greater preventive medicine. BMC Med. 2014;12(1):136. 10.1186/s12916-014-0136-z PMC4243717 25266304

[17] 17. Hamman RF, Wing RR, Edelstein SL, Lachin JM, Bray GA, Delahanty L, et al. Effect of weight loss with lifestyle intervention on risk of diabetes. Diabetes Care. 2006;29(9):2102–7. 10.2337/dc06-0560 PMC1762038 16936160

[18] 18. Miller CK, Nagaraja HN, Weinhold KR. Early weight-loss success identifies nonresponders after a lifestyle intervention in a worksite diabetes prevention trial. J Acad Nutr Diet. 2015;115(9):1464-71. 10.1016/j.jand.2015.04.022 PMC4554978 26095435

[19] 19. Kramer MK, Molenaar DM, Arena VC, Venditti EM, Meehan RJ, Miller RG, et al. Improving employee health: evaluation of a worksite lifestyle change program to decrease risk factors for diabetes and cardiovascular disease. J Occup Environ Med. 2015;57(3):284-91. 10.1097/JOM.0000000000000350 PMC4351781 25742535

[20] 20. Weinhold KR, Miller CK, Marrero DG, Nagaraja HN, Focht BC, Gascon GM. A randomized controlled trial translating the Diabetes Prevention Program to a university worksite, Ohio, 2012-2014. Prev Chronic Dis. 2015;12:150301. 10.5888/pcd12.150301 PMC4674443 26605710

[21] 21. Faghri PD, Li R. Effectiveness of financial incentives in a Worksite Diabetes Prevention Program. Open Obes J. 2014;6:1-12. 10.2174/1876823720140107001 PMC4920480 27347276

[22] 22. Zyriax BC, Letsch B, Stock S, Windler E. DELIGHT (delay of impaired glucose tolerance by a healthy lifestyle trial) - a feasibility study on implementing a program of sustainable diabetes prevention in German companies. Exp Clin Endocrinol Diabetes. 2014;122(1):20-6. 10.1055/s-0033-1355423 24132568

[23] 23. Viitasalo K, Hemiö K, Puttonen S, Hyvärinen HK, Leiviska J, Harma M, et al. Prevention of diabetes and cardiovascular diseases in occupational health care: feasibility and effectiveness. Prim Care Diabetes. 2015;9(2):96-104. 10.1016/j.pcd.2014.07.008 25128324

[24] 24. Maruyama C, Kimura M, Okumura H, Hayashi K, Arao T. Effect of a worksite-based intervention program on metabolic parameters in middle-aged male white-collar workers: a randomized controlled trial. Prev Med. 2010;51(1):11-7. 10.1016/j.ypmed.2010.04.008 20403374

[25] 25. Limaye T, Kumaran K, Joglekar C, Bhat D, Kulkarni R, Nanivadekar A, et al. Efficacy of a virtual assistance-based lifestyle intervention in reducing risk factors for Type 2 diabetes in young employees in the information technology industry in India: LIMIT, a randomized controlled trial. Diabet Med. 2017;34(4):563-8. 10.1111/dme.13258 27589695

[26] 26. Watanabe M, Yamaoka K, Yokotsuka M, Tango T. Randomized controlled trial of a new dietary education program to prevent type 2 diabetes in a high-risk group of Japanese male workers. Diabetes Care. 2003;26(12):3209-14. 10.2337/diacare.26.12.3209 14633803

[27] 27. Burton WN, Chen C-Y, Li X, Erickson D, McCluskey M, Schultz A. A Worksite Occupational Health Clinic-Based Diabetes Mellitus Management Program. Popul Health Manag. 2015;18(6):429-36. 10.1089/pop.2014.0141 26091060

[28] 28. Bevis CC, Nogle JM, Forges B, Chen PC, Sievers D, Lucas KR, et al. Diabetes wellness care: a successful employer-endorsed program for employees. J Occup Environ Med. 2014;56(10):1052-61. 10.1097/JOM.0000000000000231 25285827

[29] 29. Cochrane Training. Cochrane handbook for systematic reviews of interventions. West Sussex, UK; 2011. (Cochrane Book Series).

[30] 30. Kramer MK, McWilliams JR, Chen HY, Siminerio LM. A community-based diabetes prevention program: evaluation of the group lifestyle balance program delivered by diabetes educators. Diabetes Educ. 2011;37(5):659-68. 10.1177/0145721711411930 21918204

[31] 31. Gan Y, Yang C, Tong X, Sun H, Cong Y, Yin X, et al. Shift work and diabetes mellitus: a meta-analysis of observational studies. Occup Environ Med. 2014;72(1):72-8. 10.1136/oemed-2014-102150 25030030

[32] 32. Patel MS, Asch DA, Troxel AB, Fletcher M, Osman-Koss R, Brady J, et al. Premium-based financial incentives did not promote workplace weight loss in a 2013-15 study. Health Aff (Millwood). 2016;35(1):71-9. 10.1377/hlthaff.2015.0945 26733703

[33] 33. Ni Mhurchu C, Aston LM, Jebb SA. Effects of worksite health promotion interventions on employee diets: a systematic review. BMC Public Health. 2010;10:62. 10.1186/1471-2458-10-62 PMC2829502 20146795

