# Supplementary material for: Interventions for the prevention of risk factors and incidence of type 2 diabetes in the work environment: a systematic review
Source: Rev Saude Publica. 2019 Nov 18;53:101. doi: 10.11606/s1518-8787.2019053001084 (PMC6863112; doi:10.11606/s1518-8787.2019053001084)
Supplement: Translation

# Translation: Intervenciones para la prevención de factores de riesgo e incidencia de diabetes tipo 2 en ambiente laboral: una revisión sistemática

**Authors:** Jorge Inolopú, Nélida Hilario-Huapaya, Martín Alonso Tantaleán-Del-Águila, Yamilee Hurtado-Roca, Cesar Ugarte-Gil

**Affiliations:** I Universidad Peruana Cayetano Heredia. Instituto de Medicina Tropical Alexander von Humboldt. Lima, Perú; II Universidad Peruana Cayetano Heredia. Facultad de Salud Pública. Lima, Perú; III EsSalud. Subgerencia de Atención Preventivo Promocional y Complejidad Creciente. Lima, Perú; IV EsSalud. Instituto de Evaluación de Tecnologías en Salud e Investigación. Lima, Perú; V Universidad Peruana Cayetano Heredia. Facultad de Medicina. Lima, Perú

**Keywords:** Diabetes Mellitus Tipo 2, prevención & control, Evaluación de Resultado (Atención de Salud), Ambiente de Trabajo, Revisión

## Abstract

**OBJETIVO:**

Evaluar la efectividad de las intervenciones para la prevención de factores de riesgo e incidencia de diabetes tipo 2 en población empleada.

**MÉTODOS:**

Revisión sistemática sobre intervenciones dirigidas a población empleada adulta en riesgo de diabetes tipo 2 publicadas en Medline, Embase, Web of Science, Registro Central Cochrane de Ensayos Controlados y Lilacs. Se seleccionaron ensayos aleatorizados, estudios cuasi-experimentales y de cohortes; en idioma inglés, español y portugués; publicados entre los años 2000 al 2017. La efectividad de la intervención fue evaluada en términos de incidencia de diabetes tipo 2 y de reducción significativa del peso corporal u otro parámetro antropométrico o metabólico.

**RESULTADOS:**

Se generaron 3.024 artículos, de los cuales fueron eliminados 2.825 que no respondieron la pregunta de investigación, 130 que no evaluaron intervenciones originales, 57 fuera del ámbito laboral y dos revisiones; quedando 10 artículos seleccionados. Las intervenciones basadas en programas estructurados previamente evaluados e integrados al ámbito laboral tuvieron impacto favorable en la reducción del peso corporal y otros factores de riesgo.

**CONCLUSIONES:**

La efectividad de las intervenciones en estilo de vida para la prevención de diabetes tipo 2 debe estar basada en programas estructurados con efectividad probada y adaptados al ámbito laboral, con participación del empleador en la disposición de horarios y ambientes en el trabajo.

## INTRODUCCIÓN

Se estima que la diabetes tipo 2 (DT2) afecta a 425 millones de personas, lo cual representa el 8.8% de la población mundial adulta^1^ . Se espera que la cifra aumente a 693 millones para el año 2045^2^ . La prevención de DT2 mediante programas estructurados para promover la actividad física y dieta saludable, denominadas intervenciones en estilo de vida (IEV), han mostrado efectividad en la reducción del riesgo de DT2^3 , 4^ . El Finnish Diabetes Prevention Study (FDPS)^5^ y el Diabetes Prevention Program (DPP)^6^ proponen como indicador de éxito la reducción del 5.0 y 7.0% del peso corporal inicial, respectivamente. Sin embargo, el éxito y sostenibilidad de estas intervenciones también depende del contexto en que se desarrollan^7^ . El ámbito laboral representa un desafío para las IEV, considerando factores de riesgo conocidos tales como inadecuada alimentación^8 , 9^ y sedentarismo^10 , 11^ , sumados a otros factores poco evaluados como el estrés laboral^12^ . La prevención de la DT2 en el contexto laboral y sus complicaciones está dirigida a mantener la salud y bienestar del trabajador, además de evitar ausentismo laboral^13^ , baja productividad^14^ y gastos significativos que afectan al sistema de salud^15 , 16^ . El objetivo del estudio fue evaluar la efectividad de las intervenciones en la prevención de factores de riesgo de DT2 en población empleada.

## MÉTODOS

Fue realizada revisión sistemática sobre intervenciones dirigidas a población empleada adulta en riesgo de DT2. Se consideraron elegibles artículos originales en inglés, español y portugués, con diseño de ensayo aleatorizado, estudio cuasi-experimental o de cohorte, y publicados entre los años 2000 y 2017. Estos estudios incluyeron intervenciones para la prevención los factores de riesgo de DT2, basados o no en algún programa previamente evaluado. La población de estudio incluyó a empleados adultos con factores de riesgo de DT2 tales como sobrepeso, obesidad, prediabetes y/o síndrome metabólico. Fueron excluidos aquellos con algún tipo de diabetes al inicio de la intervención.

Se utilizó un algoritmo de búsqueda altamente sensible para las bases de datos de PubMed, Embase, Web of Science, Lilacs y en el Registro Central Cochrane de Ensayos Controlados (CENTRAL) ( Cuadro ).

**Cuadro:** Términos de Búsqueda.

•Pubmed: ((“Cohort Studies”[Mesh] OR “Clinical Trial” [Publication Type]) AND ((“2000/01/01”[Date - Publication] : “2017/30/12”[Date - Publication]))) AND (“Adult”[Mesh] OR “Occupational Groups”[Mesh] OR “Occupational Health Nursing”[Mesh] OR “Workplace”[Mesh] OR (“Occupational Groups”[Mesh] AND “Population”[Mesh]) OR Work-related[All Fields] OR Worksite[All Fields] OR Working population[All Fields] OR Economically active population[All Fields]) AND (“Occupational Health”[Mesh] OR “Primary Prevention”[Mesh] OR “Occupational Health Services”[Mesh] OR “Preventive Health Services”[Mesh] OR “Complementary Therapies”[Mesh] OR “Integrative Medicine”[Mesh] OR “Models, Organizational”[Mesh] OR “Behavior Therapy”[Mesh] OR “Tai Ji”[Mesh] OR “Yoga”[Mesh] OR “Qigong”[Mesh]) AND (“Diabetes Mellitus, Type 2” [Mesh]) AND (Diabetes Mellitus, Type 2/prevention and control[MAJR] OR “Prediabetic State”[Mesh] OR “Hyperglycemia”[Mesh] OR “Obesity, Abdominal”[Mesh] OR “Obesity”[Mesh] OR “Hypertension”[Mesh] OR “Metabolic Syndrome X”[Mesh] OR “Overweight”[Mesh] OR “Body Mass Index”[Mesh] OR “Body Weight”[Mesh] OR “Sagittal Abdominal Diameter”[Mesh] OR “Self Care”[Mesh] OR “Health Knowledge, Attitudes, Practice”[Mesh] OR “Quality of Life”[Mesh]) NOT “Diabetes, Gestational”[Mesh] NOT “Animal Experimentation”[Mesh] NOT “Diabetes Mellitus, Type 1”[Mesh]
•Embase: #1: (‘prevention’/exp OR ‘yoga’/exp OR ‘tai chi’/exp OR ‘alternative medicine’/exp OR ‘primary prevention’/exp OR ‘occupational health’/exp); #2: (‘diabetes mellitus’/exp OR ‘impaired glucose tolerance’/exp OR ‘obesity’/exp OR ‘metabolic syndrome x’/exp) AND (‘prevention’/exp OR ‘yoga’/exp OR ‘tai chi’/exp OR ‘alternative medicine’/exp OR ‘primary prevention’/exp OR ‘occupational health’/exp); #3: (‘cohort analysis’/exp OR ‘cohort analysis’ OR [cochrane review]/lim OR [systematic review]/lim OR [meta analysis]/lim OR [controlled clinical trial]/lim OR [randomized controlled trial]/lim) AND (‘diabetes mellitus’/exp OR ‘impaired glucose tolerance’/exp OR ‘obesity’/exp OR ‘metabolic syndrome x’/exp); #4: ([english]/lim OR [portuguese]/lim OR [spanish]/lim) AND [adult]/lim AND [humans]/lim AND [2000-2017]/py AND [article]/lim AND ([embase]/lim OR [embase classic]/lim); #5: #1 AND #2 AND #3 AND #4
•Web of Science: #1: (TS=(“Occupational Health” OR “Primary Prevention” OR “Occupational Health Services” OR “Preventive Health Services” OR “Complementary Therapies” OR “Integrative Medicine” OR “Models, Organizational” OR “Behavior Therapy” OR “Tai Ji” OR “Yoga” OR “Qigong”)); #2: (TS=(“diabetes” OR “Metabolic Syndrome” OR “Prediabetic State”)); #3: (TS=((“adult population” OR “Adult” OR “Occupational Groups” OR “Occupational Health Nursing” OR “Workplace” OR (“Occupational Groups” AND “Population”) OR “Work-related” OR “Worksite” OR “Working population” OR “Economically active population”))) AND LANGUAGE: (English OR Portuguese OR Spanish) AND DOCUMENT TYPES: (Article); #4: #1 AND #2 AND #3, Indexes=SCI-EXPANDED, SSCI, A&HCI, CPCI-S, CPCI-SSH, BKCI-S, BKCI-SSH, ESCI, CCR-EXPANDED, IC Timespan=2000-2017
•CENTRAL: #1: MeSH descriptor: [Diabetes Mellitus, Type 2] explode all trees and with qualifier(s): [Prevention & control - PC]; #2: MeSH descriptor: [Workplace] explode all tres; #3: #1 AND #2; Publication Year from 2000 to 2017
•Lilacs: (tw:(DIABETES)) AND (tw:(PREVENTION)) AND (tw:(WORKPLACE))

Se creó una lista de títulos y resúmenes basado en los resultados de la búsqueda en las bases de datos con un gestor de referencias bibliográficas (EndNote^TM^x8, USA). La estrategia de selección incluyó un primer tamizaje de títulos y/o resúmenes y un segundo tamizaje basado en lectura a texto completo realizado por dos de los autores (JI y NH), resolviendo las discrepancias de ambos pasos con un tercer revisor (CU-G).

La efectividad de las intervenciones consideró la incidencia de DT2 o la reducción significativa del peso corporal inicial, dado que este parámetro es reconocido como el principal predictor del riesgo de DT2^17 , 18^ , o cualquier otro parámetro antropométrico o metabólico reconocido como predictor de riesgo de DT2 ( Figura 1 ).

**Figura 1:** Marco de trabajo de la revisión sistemática.

## RESULTADOS

La búsqueda generó 3.083 artículos, de los cuales fueron eliminados 59 artículos duplicados, quedando 3.024. En el primer tamizaje, se excluyeron 2.825 artículos que no respondieron a la pregunta de investigación. En el segundo tamizaje, se excluyeron 189 artículos por tener otro diseño de estudio (n = 67), no considerar el ámbito laboral (n = 57) y no excluir participantes con DT2 (n = 39). Por tanto, se seleccionaron 10 artículos, de los cuales seis fueron ensayos aleatorizados y cuatro estudios cuasi-experimentales de tipo pre y post – test de un grupo ( Figura 2 ). Los resultados muestran heterogeneidad en el número, duración y contenido de las sesiones de las intervenciones evaluadas; así como diverso grupo etario, nivel educativo y ámbito laboral de los participantes ( Tabla 1 ).

**Figura 2:** Proceso de selección de estudios incluidos en el análisis.

**Tabla 1:** Características de los estudios sobre la prevención de factores de riesgo de DT2 en ambiente laboral.

Referencia	País	Intervención - – Programa	Diseño de estudio	Seguimiento (meses)	Factor de riesgo	Población	Ámbito laboral
Limaye et al.^25^(2016)	India	IEV 2 - NICE	Ensayo aleatorizado	12	≥ 3 factores de riesgo cardiometabólico	•265 participantes: 132 controles y 133 intervenidos, con una edad promedio de 36.2 (DE = 8.0) años siendo el 75% de sexo masculino.	Informáticos
Kramer et al.^19^(2015	USA	IEV 1 - DPP	Ensayo aleatorizado	12	Prediabetes, IMC ≥ 24, SM o hiperlipidemia	•89 participantes: 60 individuos en el primer grupo (adelantado) y 29 en el segundo (atrasado), con edad promedio de 52.3 años (rango: 34–70), siendo el 45% de sexo masculino.	Empresa farmacéutica
Weinhold et al.^20^(2015)	USA	IEV 1 - DPP	Ensayo aleatorizado	07	Prediabetes + IMC ≥ 25	•69 participantes (34 controles y 35 intervenidos) con edades entre 18 y 65 años, siendo el 20% de sexo masculino.	Empleados universitarios
Faghri et al.^21^(2014)	USA	IEV 1 - DPP	Ensayo aleatorizado	07	Score de riesgo (DRS) + IMC ≥ 25	•99 participantes: 51 en el grupo con incentivo y 48 en el grupo sin incentivo. Edad promedio del grupo sin y con incentivo: 48.98 (DE = 11.23) años y 45.14 (DE = 11.27) años respectivamente.	Técnicos en enfermería
Maruyama et al.^24^(2010)	Japón	IEV 1 - LiSM10!	Ensayo aleatorizado	04	SM	•87 participantes de sexo masculino: 49 en grupo control y 52 en grupo intervenido, con una edad promedio de 52.0 (DE = 7.9) años.	Oficinistas
Watanabe et al.^26^(2003)	Japón	Nutricional - JDS/ADA	Ensayo aleatorizado	12	Prediabetes	•173 participantes de sexo masculino: 77 en grupo control y 79 en grupo intervenido, con edad promedio de 55 años.	No especificado
Zyriax et al.^22^(2014)	Alemania	IEV 1 - FDPS	Estudio pre y post-test	36	Circunferencia abdominal elevado + prediabetes	•300 participantes con una edad promedio de 43.6. Se evalúan participantes con riesgo de DT2 y con diagnóstico de DT2 por separado.	No especificado
Viitasalo et al.^23^(2015)	Finlandia	IEV 1 - FDPS	Estudio pre y post-test	30	Score de riesgo	•1347 participantes: 945 de bajo riesgo de DT2 y 402 con alto riesgo de DT2 con edad promedio en hombres y mujeres de 50 (DE = 7.3) años y 47 (DE = 8.0) años respectivamente.	Empleados de línea aérea
Burton et al.^27^(2015)	USA	IEV 1 - HLDP	Estudio pre y post-test	12	Prediabetes	•34 participantes con prediabetes, 65 diabetes y 2 con otros factores de riesgo. Se evalúan participantes con riesgo de DT2 y con diagnóstico de DT2 por separado.	Servicios financieros
Bevis et al.^28^(2014)	USA	IEV 2 - NR	Estudio pre y post-test	12	Prediabetes	•73 participantes con prediabetes con una edad promedio de 50.0 (DE = 10.0) años, siendo el 74% de sexo masculino. Se evalúan participantes con riesgo de DT2 y con diagnóstico de DT2 por separado.	No especificado

IEV 1: intervención convencional (con enfoque nutricional y de actividad física); IEV 2: intervención convencional con monitoreo y consejería virtual; Nutricional: intervención exclusivamente nutricional; SM: síndrome metabólico; IMC: indice de masa corporal; DPP: Diabetes Prevention Program; FDPS: Finnish Diabetes Prevention Study; LiSM10!: Life Style Modification Program for Physical Activity and Nutrition Program; JDS/ADA: Japan Diabetes Society and American Diabetes Association; HLDP: Healthy Living with Diabetes Program; NR: No reportado. DE: desviación estándar. DRS: Diabetic Retinopathy Study.

Las intervenciones fueron clasificadas: siete fueron IEV convencional, dos IEV consejería virtual y una exclusivamente nutricional. De ellas, seis fueron basadas en los programas estructurados para la prevención de DT2 tales como la DPP^19 - 21^ , FDPS^22 , 23^ y el Life Style Modification Program for Physical Activity and Nutrition program (LiSM10!)^24^ . Dos intervenciones estuvieron basadas en recomendaciones para la prevención de DT2 basadas en guías de The National Institute for Health and Care Excellence (NICE)^25^ y la Japan Diabetes Society y la American Diabetes Association (JDS/ADA)^26^ . Además, una intervención estuvo basada en un programa para el manejo de la DT2 denominado Healthy Living with Diabetes Program^27^ y una no reportó antecedentes^28^ ( Tabla 2 ). La evaluación del riesgo de sesgo se describió y valoró según lo recomendado por la Colaboración Cochrane para estudios aleatorizados^29^ ( Figura 3 ).

**Tabla 2:** Resultados de los estudios sobre la prevención de factores de riesgo de DT2 en ambiente laboral.

Referencia	País	Intervención - estructura	Incentivo	Rol del empleador	Resultado	Valoración
Limaye et al.^25^(2016)	India	1 sesión grupal al inicio y cada 3 meses.	NR	Enrolamiento	•Reducción significativa del peso corporal, IMC y circunferencia abdominal respecto al grupo control. Se reporta reducción de la prevalencia de IMC ≥ 25 kg/m^2^del 6.0 % en el grupo intervenido y aumento del 6.8% en el grupo control. •No se reporta reducción del porcentaje de peso perdido.	Efectivo
Kramer et al.^19^(2015 )	USA	12 sesiones semanales por 3 meses, 4 bisemanales por 3 meses y 6 mensuales de mantenimiento por 6 meses.	Tarjetas de descuento.	Ambiente y horarios en el trabajo	•Reducción significativa del peso corporal, HbA1C, perímetro abdominal e IMC entre los grupos de estudio a los 6 meses de intervención. •Reducción del 5% de peso corporal inicial en el 45% en el primer grupo y 7% en el segundo grupo a los 6 meses de intervención.	Efectivo
Weinhold et al.^20^(2015)	USA	16 sesiones semanales por 4 meses.	NR	Ambiente y horarios en el trabajo	•Reducción significativa del peso, IMC, circunferencia abdominal, niveles de glucosa en ayunas en el grupo intervenido y respecto al grupo control. •En el grupo intervenido y control, el 52.9% y 2.9% de los participantes logró perder al menos 5% del peso corporal respectivamente post-intervención.	Efectivo
Faghri et al.^21^(2014)	USA	16 sesiones de 60 minutos por semana durante 4 meses.	Incentivo económico acumulable.	Enrolamiento	•Reducción significativa del peso e IMC en el grupo con incentivo económico respecto al grupo sin incentivo económico. •No se reporta reducción del porcentaje de peso perdido.	Efectivo
Maruyama et al.^24^(2010)	Japon	1 sesión al inicio de la intervención, consejería individualizada en 2 meses y 2 meses siguientes mediante internet.	NR	Enrolamiento	•Reducción significativa de 14 de 17 parámetros que definen al riesgo de desarrollar DT2, entre los cuales se encuentra peso e índice de masa corporal. •No se reporta reducción del porcentaje de peso perdido.	Efectivo
Watanabe et al.^26^(2003)	Japon	1 sesión al mes del estudio 1 sesión al sexto mes del estudio vía e-mail.	NR	No especificado	•Reducción significativa de ingesta calórica diaria, especialmente horarios nocturnos, con subsecuente disminución de los niveles de glucosa post-pandrial a las 2 horas respecto al grupo control. •No se reporta reducción del porcentaje de peso perdido.	Efectivo
Zyriax et al.^22^(2014)	Alemania	6 sesiones mensuales por 6 meses, 6 sesiones bisemanales por 6 meses y 1 sesión trimestral por 2 años.	NR	Enrolamiento	•Reducción significativa del peso, índice de masa corporal y circunferencia abdominal. En los hombres y mujeres hubo una reducción del 4.3 ± 3.6 y 3.6 ± 2.4 kg, respectivamente post-intervención. •No se reporta reducción del porcentaje de peso perdido.	Efectivo
Viitasalo et al.^23^(2015)	Finlandia	No especificada.	NR	Enrolamiento	•Existió incremento del riesgo de DT2, mientras que en el grupo con alto riesgo la reducción del riesgo de DT2 fue no significativa. •Reducción del ≥ 5% fue reportado en el 20.8% de los hombres y 7.0% de mujeres que asistieron a más de una sesión.	No efectivo
Burton et al.^27^(2015)	USA	No especificada.	Glucómetros e incentivo económico al completar estudio.	Programa de bienestar	•No se mostró reducción significativa del Índice de Masa Corporal (IMC) durante el estudio, el cual se mostró en niveles mayores de 30 kg/m^2^durante el estudio. •No se reporta reducción del porcentaje de peso perdido.	No efectivo
Bevis et al.^28^(2014)	USA	4 sesiones educativas de 2 horas en el primer semestre y 8 sesiones telefónicas siguientes.	Glucómetros y afiliación en programa de bienestar.	Programa de bienestar	•No se mostró reducción significativa del IMC, el cual fue mayor de 35 kg/m^2^durante el estudio, ni hubo reducción significativa de los niveles de Hemoglobina glicosilada (HbA1c). •No se reporta reducción del porcentaje de peso perdido.	No efectivo

NR: no reportado; IMC: indice de masa corporal; DT2: diabetes tipo 2; HbA1c: hemoglobina glicosilada

**Figura 3:** Resumen de la valoración del riesgo de sesgo de los estudios aleatorizados según la Colaboración Cochrane.

De acuerdo a la valoración de la efectividad, seis estudios mostraron reducción estadísticamente significativa del peso corporal^20^ , los cuales estuvieron basados en programas estructurados para la prevención de DT2 tales como la DPP^19^ , FDPS^22^ y LiSM10!^24^ , así como en las recomendaciones dadas por la NICE^25^ . Por otro lado, una intervención de tipo nutricional mostró una reducción de la ingesta calórica con subsecuente reducción de los niveles de glucosa post-prandrial de dos horas basado en las recomendaciones de la JDS/ADA^26^ . En contraste, dos intervenciones no efectivas tuvieron un enfoque dirigido al manejo de la DT2^27 , 28^ y una tuvo limitadas sesiones y prolongado seguimiento^23^ . Ningún estudio mostró efectos adversos en la población de estudio.

Ninguno de los siete estudios efectivos mostró resultados en términos de reducción del riesgo de incidencia de DT2 dado que su diseño no lo permitió. Sin embargo, dos de ellos mostraron resultados en términos de porcentaje de reducción del peso corporal inicial^19 , 20^ . Kramer et al.^19^ determinaron que la reducción del promedio de peso corporal de 5.0% fue logrado por el 45.0% y el 7.0% de los participantes intervenidos y del grupo control, respectivamente^19^ . Por su parte, Weinhold et al.^20^ determinaron que el 32.4% del grupo intervenido logró perder al menos un 7.0% del peso corporal inicial, lo cual fue significativamente mayor que el grupo control (2.9%; p < 0.01).

Por otro lado, se evaluaron tales como los incentivos económicos y no económicos, al igual que el rol que cumple la participación del empleador en el resultado de la intervención. Faghri et al.^21^ fue el único estudio en ofrecer un incentivo económico acumulable por peso perdido. A pesar de ser una intervención de baja intensidad, tuvo éxito en reducir peso, disminuir el riesgo de DT2 y tener una alimentación saludable. La participación del empleador, generalmente no fue más allá en el apoyo en el enrolamiento de los participantes. Las facilidades en el enrolamiento, disposición de ambientes y horarios de trabajo en los estudios de Kramer et al.^19^ y Weinhold et al.^20^ estuvieron vinculados al éxito de la intervención.

## DISCUSIÓN

Las intervenciones basadas en programas estructurados para la prevención de DT2 mostraron amplia efectividad y ello estuvo vinculado al grado de similitud con el programa base. Por su parte, las intervenciones basadas en recomendaciones de las guías NICE fueron efectivas estadísticamente, pero presentan aspectos a ser considerados. El estudio de Limaye et al.^25^ estuvo basado en la guía NICE y muestra reducción significativa del peso, pero esta es lograda debido a un aumento del peso en el grupo control. En este estudio, el promedio de reducción promedio del peso en el grupo intervenido no llega ser menor del 5.0% post-intervención.

Las intervenciones que no muestran efectividad^23 , 27 , 28^ no presentaran un plan estructurado de sesiones^23^ y dos de ellas estuvieron enfocadas al manejo de DT2^27 , 28^ . Viitasalo et al.^23^ en un estudio pre y post - test realizado en empleados de una compañía aérea, mostraron un incremento ligero en los parámetros antropométricos luego de la intervención. Esta tuvo un promedio de 1.6 sesiones, la cual es menor a cualquier otro programa existente; aunque basado en la FDPS, el programa difirió del original. En cuanto a las dos intervenciones dirigidas al manejo de DT2, estas incluyeron a población con prediabetes en un contexto de trabajadores con DT2. Este tipo de intervenciones estuvieron basadas en los programas de bienestar laboral y a pesar de disponer de recursos, el enfoque de la intervención es importante.

En general, los artículos seleccionados hacen una breve descripción de los factores laborales que favorecerían el desarrollo de DT2. Limaye et al.^25^ evaluaron una intervención en empleados informáticos en India quienes reportaran un estilo de vida sedentario, inadecuados hábitos alimenticios y estrés laboral. Maruyama et al.^24^ evaluaron a oficinistas japoneses con periodos prolongados de trabajo que favorecerían una elevada ingesta calórica. Por su parte, Watanabe et al.^26^ , evaluaron a trabajadores con condiciones de trabajo relacionados a alta ingesta calórica nocturna y cortos periodos de sueño.

Los empleados que realizaban estas actividades concuerdan con las labores de los “white-collar workers”. Esta población tiene mayor riesgo de presentar sobrepeso y obesidad debido al sedentarismo que experimentan en el trabajo, favoreciendo el incremento del índice de masa corporal^10^ . Por otro lado, trabajos con turnos nocturnos, rotativos, o no regulares, denominados “shift work” están asociados al desarrollo de DT2 debido a las insuficientes horas de sueño y malos hábitos alimenticios^31^ . No obstante, estos factores no fueron evaluados en la intervención, pero debieron ser considerados en la evaluación de su impacto.

La participación del empleador en el enrolamiento se dio mediante llamadas telefónicas y correos electrónicos^21 , 23 - 25^ . Otros estudios además desarrollan planes de comunicación para incentivar cambios en el estilo de vida^19 , 20 , 22 , 27 , 28^ e incluso brindan ambientes y horarios para el desarrollo de las sesiones en el trabajo^19 , 20 , 27^ . Algunas intervenciones brindan incentivos básicos como glucómetros^27 , 28^ , tarjetas de descuento al iniciar y completar la intervención^19 , 27^ e incentivos económicos por peso perdido^21^ . Sin embargo, los resultados a largo de plazo en el mantenimiento del peso perdido por este tipo de intervenciones parecen no ser consistentes^32^ .

Aquellos empleadores que ofrecieron mayor apoyo en la intervención, tales como facilidades en el enrolamiento, disposición de ambientes (tópico, ambientes de descanso, cafeterías, etc.) y horarios en el trabajo favorecieron la implementación y el éxito de la intervención^19 , 20^ . Estos beneficios pueden integrase la intervención a fin de lograr y mantener los objetivos propuestos. Las empresas con servicio de alimentación pueden implementar opciones de menú saludable para personas en riesgo de DT2 e incluso diabéticos^33^ . En nuestra revisión, las intervenciones que no reportan la presencia de un programa con tales beneficios son aquellas orientadas principalmente a los “white-collar workers”^24 - 26^ .

Las limitaciones de nuestra revisión están vinculadas a la heterogeneidad de las intervenciones que imposibilitan el meta-análisis. Por otro lado, no se cuenta con resultados en términos de riesgo de incidencia de DT2 debido a los periodos cortos de intervención de la mayoría de estudios evaluados. Así mismo, la valoración de la efectividad de las intervenciones es flexible dado que se basan en la reducción estadísticamente significativa del peso corporal (p < 0.05), mas no en la reducción del porcentaje de peso corporal o en una reducción de peso que tenga un significado clínico (reducción de al menos 5.0% post-intervención). Además, el riesgo de sesgo de los ensayos aleatorizados es elevado. Los estudios de Weinhold et al.^20^ y Watanabe et al.^26^ muestran mayor proporción de riesgo poco claro pero con mejor valoración, mientras que el artículo de Kramer et al.^19^ tiene la más baja valoración.

Las intervenciones con impacto favorable en la reducción del peso corporal y otros factores de riesgo de DT2 en el ámbito laboral son principalmente aquellas basadas en un programa previamente evaluado y realizadas en el centro laboral con participación del empleador. Se requieren estudios longitudinales que evalúen dichas intervenciones como potenciales programas para reducir la incidencia de DT2 en el ámbito laboral.
